# Occurrence and human exposure assessment of bisphenol analogues in various paper products from Korea

**DOI:** 10.3389/fpubh.2026.1748104

**Published:** 2026-02-04

**Authors:** Mangong Shin, Jae-Eun Lim, Sori Mok, Chunyang Liao, Hyo-Bang Moon

**Affiliations:** 1 Department of Marine Science and Convergence Technology, College of Engineering Sciences, Hanyang University, Ansan, Republic of Korea; 2 State Key Laboratory of Environmental Chemistry and Ecotoxicology, Research Center for Eco-Environmental Sciences, Chinese Academy of Sciences, Beijing, China

**Keywords:** bisphenol S, BPA, business-type differences, dermal exposure, thermal receipt

## Abstract

Bisphenol A (BPA) has been widely used in various paper products, particularly as a color developer in thermal receipts. Growing concerns about its toxicity have prompted the introduction of other bisphenols as alternatives. This study investigated the occurrence and residue levels of BPA and its analogues in thermal paper receipts (*n* = 120) and other paper products (*n* = 32) collected in Korea in 2015, with a particular focus on business-type-specific differences during an early phase of BPA substitution. BPA was the dominant compound in most receipts, while bisphenol S (BPS) was the most common substitute in “BPA-free” labeled receipts. Total concentrations of bisphenol analogues in thermal receipts were 2–4 orders of magnitude higher than those in other paper products. Multivariate analysis identified distinct compositional clusters associated with different color developers. Large retail chains showed a higher prevalence of BPS-based receipts, whereas small local stores predominantly relied on BPA-based thermal papers, indicating business-type-specific differences in substitution timing. Dermal exposure estimates indicated that occupational groups, particularly workers in small local stores, experienced substantially higher exposure than the general population. Estimated BPA intakes via paper handling exceeded the revised tolerable daily intake proposed by the European Food Safety Authority, highlighting the need for comprehensive exposure assessments of bisphenol analogues, especially in occupational settings.

## 1 Introduction

Bisphenol A (BPA) has been widely used as a starting material in various industrial and consumer products, including water pipes, electronic equipment, toys, and thermal papers (1–3). In 2012, the European Thermal Paper Association reported that BPA was used in 80% of the thermal papers in European Union (EU) (4) and BPA was detected in 100% of thermal paper receipts in the United States (US), Vietnam, and China (5, 6). Concerns about the potential toxicity of BPA have grown, particularly regarding its adverse effects on the reproductive system, fetal development, and its role as an endocrine disruptor (7, 8). BPA has been detected in various biological matrices, such as serum, urine, placenta, breast milk, and umbilical cord serum, indicating potentials for health risks (9, 10). Previous studies have reported elevated BPA levels in the urine of cashiers frequently handling thermal paper receipts compared to the general population (1, 11–13). To mitigate such risks, the European Commission implemented a regulation in April 2016 prohibiting thermal paper containing ≥0.02% BPA by weight from being placed on the market from January 2020 (14). Following this precedent, Korean government introduced a comparable restriction in 2021, with full enforcement scheduled from April 2024, thereby aligning its policy with international efforts to phase out BPA in thermal papers (15).

In response to increasing concerns over BPA toxicity, bisphenol S (BPS) has been introduced as a primary substitute color developer in thermal papers. Other bisphenols, including bisphenol F (BPF), bisphenol AF (BPAF) and bisphenol B (BPB), have occasionally been detected in thermal papers; however, these compounds are generally present at trace levels and are not considered intentional color developers (6, 16–18). Nevertheless, many bisphenol analogs, such as BPS, share structural similarities with BPA and have been reported to exhibit comparable endocrine-disrupting activities (19). BPS, the most widely used substitute, is of particular concern due to its reproductive and endocrine-disrupting toxicity (20, 21). Moreover, BPS has been classified as a substance of very high concern in the EU (22). Although BPS exhibits lower dermal penetration than BPA (23–25), biomonitoring studies have observed elevated urinary BPS concentrations in cashiers handling BPS-containing thermal paper, confirming dermal absorption as a significant exposure pathway (26). Thus, the replacement of BPA with other bisphenols may not substantially reduce associated health risks to humans.

The choice of color developers in thermal paper has shifted over time according to the regulatory actions (27, 28). A few studies have suggested that usage patterns may also vary by business type (27, 29). However, most risk assessments have classified exposure only by general vs. occupational groups (5, 6, 30, 31), without considering variation by business type. This represents a notable gap, as differences in bisphenol use between large retail chains and small local stores could result in differing exposure profiles and health risks. To address this, the present study analyzed bisphenol analogs in thermal paper receipts and various paper products collected in Korea, providing a snapshot of market heterogeneity during the early phase of BPA substitution, with a focus on business-type-specific differences in substitution timing. This approach enables a more precise assessment of exposure variability across business types during this transitional period.

## 2 Materials and methods

### 2.1 Sample collection

Thermal receipts were collected randomly from various places (e.g., cafes, restaurants, department stores, hospitals, banks) from Korea in 2015, and then classified into three categories based on the business type: large retail chains (*n* = 41; e.g., franchise supermarkets and department stores), small local stores (*n* = 59; e.g., small local restaurants or local café), and public service facilities (*n* = 20; e.g., government offices, hospitals, and public transportation). Large retail chains refer to national or regional franchise-based supermarkets and department stores that handle a high volume of transactions. Small local stores include individually owned or small-scale shops which typically operate with limited staff and may not follow standardized printing or handling protocols. Public service facilities were grouped based on their roles in providing essential services frequently accessed by the general public, regardless of ownership. In addition to thermal receipts, other types of papers were also collected, including newspapers (*n* = 5), business cards (*n* = 5), café coupons (*n* = 5), airplane boarding passes (*n* = 6), and paper currencies (*n* = 11). All collected samples were individually placed in polyethylene bags, which were pre-checked to confirm the absence of target compounds, and kept in the dark until further processing.

### 2.2 Standards and reagents

The structures of target bisphenol analogs are shown in Supplementary Table S1. Analytical reference standards of BPA (CAS 80-05-7, 98%), BPS (CAS 80-09-1, 98%), BPF (CAS 620-92-8, 98%), bisphenol AP (BPAP, CAS 1571-75-1, 98%), bisphenol P (BPP, CAS 2167-51-3, 98%), BPAF (CAS 1478-61-1, 98%), and bisphenol Z (BPZ, CAS 843-55-0, 98%) were purchased from Cambridge Isotope Laboratories (Andover, MA, USA). For quantification, ^13^C_12_-BPA (99%) and ^13^C_12_-BPS (98%) were used as internal standards, both obtained from Cambridge Isotope Laboratories. Anhydrous sodium sulfate (Na_2_SO_4_) was supplied by Kanto Chemical Co., Inc. (Tokyo, Japan). High performance liquid chromatography (HPLC) grade methanol was supplied by J.T. Baker (Phillipsburg, NJ, USA). Ultrapure water was obtained using a Milli-Q Reference Water Purification System (Merck Millipore, Burlington, MA, USA).

### 2.3 Sample preparation

Samples were extracted following a previously published method, with slight modifications (5, 30, 32). Circular spots were punched from the center area of each sample, and a total of approximately 20 mg was collected per sample. The punched pieces were transferred into pre-washed 15 mL polypropylene (PP) tube and extracted with 5 mL of methanol by shaking for 30 min. The mixture was then centrifuged at 3,500 rpm for 5 min, and the supernatant was transferred to another PP tube. The extraction was repeated once more with 5 mL of methanol. Both extracts were combined, and an aliquot was diluted 1:1,000 with methanol. The diluted extract was concentrated to approximately 1 mL under a gentle stream of nitrogen, spiked with 40 ng of internal standards, filtered through a 0.22 μm nylon filter (Restek Corp., Bellefonte, PA, USA) for instrumental analysis.

### 2.4 Instrumental analysis

HPLC (Agilent 1260 infinity, Agilent Technologies, Wilmington, DE, USA) coupled with an API 4500 electrospray triple quadrupole mass spectrometer (MS/MS; AB Sciex, Applied Biosystems, Foster City, CA, USA) was used for determination of bisphenol analogs. Detailed instrumental conditions of HPLC-MS/MS were similar to those reported earlier (2, 33, 34). Briefly, a Betasil C_18_ column (100 mm × 2.1 mm, 5 μm; Thermo Fisher Scientific, Munich, Germany) was used for separation of target compounds. The mobile phase flow rate was 200 μL/min, and the injection volume was 10 μL. The mobile phase were 90:10 (v/v) mixture of water and methanol (A) and methanol (B), operated under the following gradient condition: 15% methanol at 0 min (held for 1 min); increased to 50% methanol from 1 to 6 min; increased to 95% methanol from 6 to 7.5 min (held for 6.5 min); and returned to 15% methanol from 14 to 15 min, held for 15 min until the next injection. Information on the multiple reaction monitoring (MRM) transitions and optimized potentials is summarized in Supplementary Table S2.

### 2.5 Quality control

Procedural blanks were prepared using anhydrous sodium sulfate and were included every 15 samples to assess background contamination throughout all experimental procedures. Trace levels of BPA (mean: 0.37 μg/g), BPS (0.02 μg/g), BPAP (0.06 μg/g), and BPZ (0.31 μg/g) were found in procedural blanks and subtracted from the measured concentrations in real samples. To evaluate matrix effects, thermal receipt confirmed to be free of all seven target compounds was spiked with known amounts of analytes (6, 18). After evaporation at room temperature, the samples were subjected to the same extraction procedure as the real samples. Matrix effects were evaluated at two concentration levels (10 ng/mL and 100 ng/mL). At 10 ng/mL, the recoveries for matrix effect test ranged from 94% ± 1.0% for BPZ to 106% ± 0.6% for BPAF. At 100 ng/mL, the recovery range was from 94% ± 0.5% for BPS to 113% ± 7.7% for BPP, except for BPAF (67% ± 11%). The lowest calibration point of each compound was used as the limit of quantification (LOQ), which ranged from 0.5 ng/g to 25 ng/g. The regression coefficients (R^2^) of the calibration curves, constructed from 13 calibration points (0.01 to 100 ng/mL), were all above 0.99. Quality control results, including matrix-spiked recoveries and LOQs, are summarized in Supplementary Table S3.

### 2.6 Data analysis

For the calculation of total bisphenol analogs (ΣBP; the sum of seven bisphenol analogs), concentrations below the LOQ were treated as zero to avoid overestimation. For statistical analysis, the concentrations of individual compound below the LOQ were substituted with LOQ/2. Group comparisons were performed using the Mann–Whitney *U*-test in SPSS version 27 (IBM Corp., Armonk, NY, USA), with a statistical significance at *p* < 0.05. Non-metric multidimensional scaling (n-MDS) and cluster analysis were performed using PRIMER for Windows (Version 5.2.9, Plymouth, UK). Prior to analysis, bisphenol concentration data were fourth-root transformed to reduce the influence of dominant compounds. Similarity between samples was calculated using the Bray-Curtis similarity index. Hierarchical cluster analysis was then conducted based on the similarity matrix using group-average linking. Clusters were defined by applying a similarity threshold of 60%, which was selected to provide interpretable grouping of samples based on their compositional similarity.

### 2.7 Estimation of dermal exposure dose via handling papers

To estimate dermal absorption of bisphenol analogs through handling papers, dermal exposure dose (DED; ng/day) was calculated using the following equation provided by Liao and Kannan (5):


$$\begin{array}{l} DED=k\;\times C\;\times HF\;\times HT\;\times AF/10^{6} \end{array}$$

where *k* is paper-to-skin transfer coefficient (21,522.4 ng/s for all bisphenol analogs), C is the concentration of total bisphenols in paper samples (μg/g), HF is handling frequency (times/day), HT is the handling time (s), and AF is the dermal absorption fraction.

Previous studies have reported a range of dermal absorption fractions for BPA, depending on experimental conditions and skin types. Biedermann et al. (35) reported an absorption fraction of 27%, while Demierre et al. (36) reported values below 10% based on experiments using the dorsal skin of the upper leg, and Mørck et al. (37) reported an absorption fraction of approximately 13% without specifying the skin site. More recently, Reale et al. (25), using human abdominal skin, reported dermal absorption fractions of approximately 25% for BPA, comparable to earlier estimates, and substantially lower absorption for BPS (0.4%). In the present study, dermal absorption fractions reported by Reale et al. (25) were adopted, as they are consistent with earlier BPA estimates and provide compound-specific information for both BPA and BPS under comparable experimental conditions. Accordingly, an absorption fraction of 25% was applied for BPA, whereas a substantially lower fraction (0.4%) was used for BPS. For other bisphenol analogs, for which compound-specific dermal absorption data are currently unavailable, the BPA absorption fraction was conservatively applied.

Handling frequency was assumed to be 2 and 150 times/day for thermal receipts, and 2 and 20 times/day for paper currencies, representing the general population and occupationally exposed individuals, respectively (5, 32). For other paper types, handling frequencies were assumed to be 5 times/day for business cards, airplane boarding passes, and café coupons (5), and 15 times/day for newspapers (38), for both exposure groups. Handling time was set at 60 seconds for newspapers (38) and 5 seconds for all other paper types (5). A detailed list of parameter values used in the calculations is provided in Supplementary Table S4.

## 3 Results and discussion

### 3.1 Occurrence and residue levels of bisphenol analogs in thermal receipts

Concentrations of bisphenol analogs measured in thermal receipts are summarized in Table 1 and Supplementary Figure S1. The ΣBP concentrations in all thermal receipt papers ranged from < LOQ to 16,900 μg/g, with a mean value of 2,350 μg/g. Five samples collected from convenience stores, department stores, and train stations showed no detectable levels of any bisphenol analogs, resulting in a detection frequency of 96% for ΣBP. Among the target compounds, BPA was the dominant analog, with a mean concentration of 2,200 μg/g and a maximum of 16,800 μg/g, detected in 88% of all samples. This finding suggests that a high contamination level by ΣBP is primarily attributable to BPA. BPS followed as the second most abundant analog, with a mean concentration of 141 μg/g and a maximum of 1,520 μg/g, although it was detected in only 15% of all samples. In contrast, other analogs were detected at substantially lower mean concentrations (< 10 μg/g), with detection frequencies ranging from 4.2% (BPAP) to 51% (BPP), indicating their relatively minor consumption compared to BPA and BPS. Among the target compounds, BPA and BPS were the only bisphenols detected at concentrations consistent with effective thermal color development (>1.00 mg/g) in receipts. The remaining bisphenols were detected at substantially lower concentration (< LOQ−64.0 μg/g) and therefore interpreted as minor bisphenols, likely originating from impurities, secondary constituents, or background contamination rather than intentional use as color developers (17). Despite the low concentration of BPP in thermal receipts, it showed unusually high detection frequency (51%). This may suggest its formation as an impurity during BPA synthesis (39), as illustrated by a possible reaction pathway in Supplementary Figure S2.

**Table 1:** Occurrence and concentrations of bisphenol analogs (μg/g) in thermal receipt.

**Compounds**	**BPA**	**BPS**	**BPF**	**BPAP**	**BPP**	**BPAF**	**BPZ**	**ΣBP**
**Thermal receipt papers**								
DF^a^ (%)	88	15	13	4.2	51	5.8	47	96
Mean	2,200	141	1.90	0.13	0.11	0.18	7.02	2,350
Median	2,100	<LOQ^b^	<LOQ	<LOQ	<LOQ	<LOQ	<LOQ	2,100
Max	16,800	1,520	32.2	4.85	1.53	9.71	64.0	16,900
SD^c^	2,200	367	5.52	0.66	0.23	1.01	9.10	2,090

^a^DF, detection frequency; ^b^LOQ, limit of quantification; ^c^SD, standard deviation.

Clear differences were observed in the detection patterns and concentrations of bisphenol analogs according to business type (Table 2). In large retail chains, ΣBP concentrations ranged up to 4,750 μg/g with a mean of 1,280 μg/g, driven largely by BPA (mean 1,080 μg/g; detection frequency of 78%), while BPS was occasionally detected at moderate levels (mean 185 μg/g; detection frequency of 22%). In contrast, small local stores exhibited the highest ΣBP concentrations, with levels reaching up to 16,900 μg/g (mean: 2,960 μg/g). This elevated burden was primarily driven by consistently high BPA concentrations (mean: 2,890 μg/g; detection frequency: 97%). Although BPS was detected less frequently (mean: 61.1 μg/g; detection frequency: 6.8%), its maximum concentration was still relatively high, reaching 1,520 μg/g. For public service facilities, ΣBP concentrations were also relatively higher (mean 2,740 μg/g), largely driven by BPA (mean 2,450 μg/g; detection frequency of 85%). However, BPS was more frequently detected (detection frequency: 25%) with a mean concentration (287 μg/g) higher than those found in small local stores.

**Table 2:** Occurrence and concentrations of bisphenol analogs (μg/g) in thermal receipt according to business type.

**Compounds**	**BPA**	**BPS**	**BPF**	**BPAP**	**BPP**	**BPAF**	**BPZ**	**ΣBP**
**Large retail chains (*****n*** = **41)**								
DF^a^ (%)	78	22	9.8	7.3	46	12	37	90
Mean	1,080	185	1.26	0.19	0.09	0.37	5.53	1,280
Median	555	<LOQ^b^	<LOQ	<LOQ	<LOQ	<LOQ	<LOQ	922
Max	4,700	1,100	16.9	3.32	0.71	9.71	35.3	4,750
SD^c^	1,440	384	3.97		0.17	1.55	8.20	1,350
**Small local stores (*****n*** = **59)**								
DF (%)	97	6.8	15	3.4	56	3.4	53	100
Mean	2,890	61.1	2.17	0.13	0.13	0.10	8.01	2,960
Median	2,900	<LOQ	<LOQ	<LOQ	0.04	<LOQ	10.5	2,910
Max	16,800	1,520	26.7	4.85	1.53	4.52	64.0	16,900
SD	2,300	271	5.54	0.73	0.28	0.62	10.2	2,250
**Public service facilities (*****n*** = **20)**								
DF (%)	85	25	10	0	45	0	50	95
Mean	2,450	287	2.43	-^d^	0.08	-	7.11	2,740
Median	2,160	<LOQ	<LOQ	-	<LOQ	-	6.06	2,160
Max	8,100	1,330	32.2	-	0.37	-	17.2	8,120
SD	2,340	514	7.89	-	0.12	-	7.37	2,080

^a^DF, detection frequency; ^b^ LOQ, limit of quantification; ^c^ SD, standard deviation; ^d^-, not reported for compounds with no detection.

The concentrations of BPA and BPS, identified as the main color developers in this study, are compared to those reported in thermal receipts from other countries during similar sampling periods (Supplementary Figure S3). The mean concentration of BPA (2,200 μg/g) in thermal receipts collected in our study was similar to those previously reported in Korea during 2010–2011 (1,560 μg/g), and higher than those reported in Albany, USA during 2010–2011 (341 μg/g), as well as in Japan, where BPA was not detected in receipt samples (5). However, the BPA concentrations observed in our study were 3–7 times lower than those reported in Guangzhou, China in 2013 (13,900 μg/g) (38), Switzerland in 2014 (13,500 μg/g) (40), Denmark in 2014 (8,300 μg/g) (41), and Vietnam during 2010–2011 (6,320 μg/g) (5). The mean concentration of BPS (141 μg/g) in thermal receipts collected in our study was considerably higher than those reported in Vietnam (0.30 μg/g) and a previous Korean study (0.70 μg/g) conducted during 2010–2011 (30). This suggests an increasing trend in BPS usage in Korea. However, it was lower than the concentrations reported in Denmark in 2014 (210 μg/g) (41), Albany, USA in 2010–2011 (443 μg/g), Japan in 2010–2011 (624 μg/g) (30), and Switzerland in 2014 (10,200 μg/g) (40). These findings suggest that Korea was in a transitional phase in 2015, during which the substitution of BPA with BPS had begun but was not yet widely implemented in thermal paper manufacturing. Accordingly, the global comparison highlights the need for continued monitoring of bisphenol analogs in thermal receipts.

Within thermal receipts, BPA and BPS concentrations exhibited a significant negative correlation, consistent with inverse relationships previously reported in thermal paper samples (Supplementary Figure S4) (30). Receipts labeled as “BPA-free” (*n* = 15) predominantly contained BPS (776–1,520 μg/g), while only trace levels of BPA were occasionally detected. The detected BPA concentrations in these samples (13.1–71.0 μg/g) were substantially lower than those observed in BPA-dominant receipts and are therefore likely attributable to background contamination or transfer during handling processes. Given that this relationship has been widely documented, it is presented here as contextual information rather than as independent evidence of substitution.

### 3.2 Differences in bisphenol usage patterns

**Figure 1:**
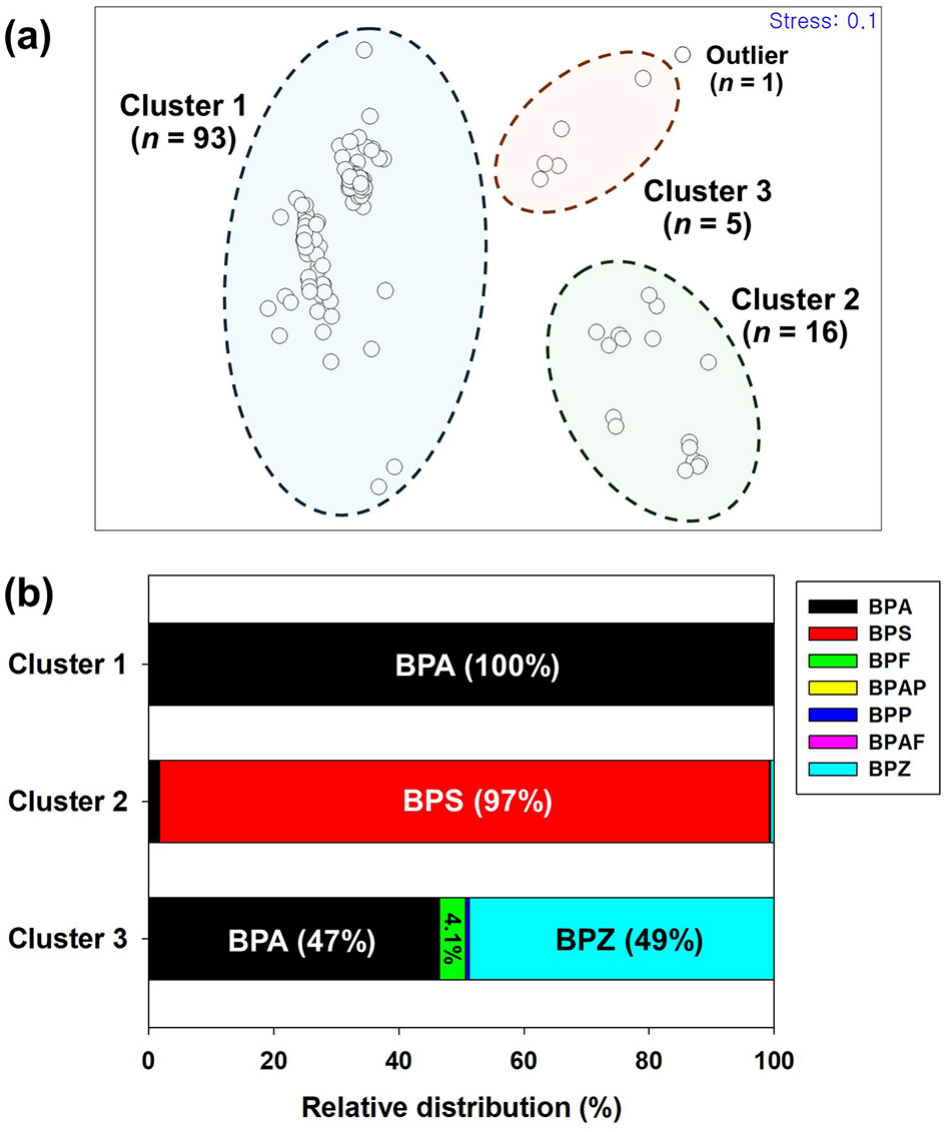
**(a)** Non-metric multidimensional scaling (n-MDS) ordination of thermal receipt samples based on bisphenol analog composition, showing four distinct clusters; **(b)** relative compositional profiles (%) of bisphenol analogs within each cluster.

To characterize the usage patterns of bisphenol analogs in thermal receipts, n-MDS analysis was conducted based on their compositional profiles. Five samples showing no detectable levels of any bisphenol analogs were excluded from further analysis. The result revealed three distinct clusters, except for one sample, and their relative compositional profiles in the samples are shown in Figure 1 and Supplementary Table S5. The largest group, cluster 1 (*n* = 93), consisted predominantly of samples containing BPA as the main color developer, indicating that BPA is still widely used in thermal receipts. This finding is consistent with the high detection frequency and the highest mean concentration of BPA described above in the overall sample set. In Korea, restrictions on BPA in thermal papers had not yet been implemented at the time of sampling, and thus in 2015 most receipts still relied on BPA-based formulations. Cluster 2 (*n* = 16) was characterized by a high proportion of BPS, and notably, 15 out of these 16 samples were labeled as “BPA-free” receipts, supporting the substitution of BPA with BPS in certain products. Cluster 3 (*n* = 5) exhibited more unusual compositions, with BPA co-occurring with BPZ and BPF. Although BPZ and BPF were detected in a small number of samples, their concentrations were far below levels for thermal color development. These findings suggest that their presence reflects background contamination rather than intentional substitution. One sample, which was collected from a department store belonging to the large retail chain group, did not fall into any of the three clusters. It contained BPF and BPP at concentrations of 16.9 μg/g and 0.28 μg/g, respectively.

To investigate potential differences in bisphenol usage patterns according to business type, the thermal receipt samples were categorized into three market groups, such as large retail chains, small local stores, and public service. The distribution of the three n-MDS derived clusters was analyzed according to market category (Supplementary Figure S5). Cluster 1, which was predominantly composed of BPA-based samples, was largely associated with small local stores (59%), suggesting that BPA remains the dominant color developer in these establishments. In contrast, cluster 2, characterized by a high proportion of BPS and mostly consisting of “BPA-free” receipts, was primarily linked to large retail chains (50%). This pattern implies that the substitution of BPA with BPS may have begun earlier and progressed more rapidly in larger retails. One plausible explanation for this trend is that larger retailers may be more responsive to consumer awareness and demand safer alternatives, or more likely to comply with voluntary or international regulatory standards. In addition, large franchises may place greater emphasis on product safety and brand reputation, which could facilitate a faster transition to alternative materials. In contrast, small local stores may be constrained by limited supplier options and/or experience less pressure from consumers, resulting in the continued use of BPA-based thermal receipts. Cluster 3, which exhibited co-occurrence of BPA with BPZ and BPF, was also mainly comprised of large retailers (80%). However, the overall bisphenol concentrations in cluster 3 were significantly (*p* < 0.05) lower than those found in clusters 1 and 2, by factors of 30 and 80 times, respectively (Supplementary Figure S6). This indicates that these receipts were based on other color developers not targeted in the present study. Previous studies have demonstrated that certain thermal receipts employ alternative color developers that were not included in our targeted compound list (6, 16, 17). In the case of public service facilities, samples were distributed relatively evenly between clusters 1 and 2, suggesting the concurrent use of both BPA- and BPS-based thermal papers in this sector.

### 3.3 Concentrations of bisphenol analogs in other paper products

**Figure 2:**
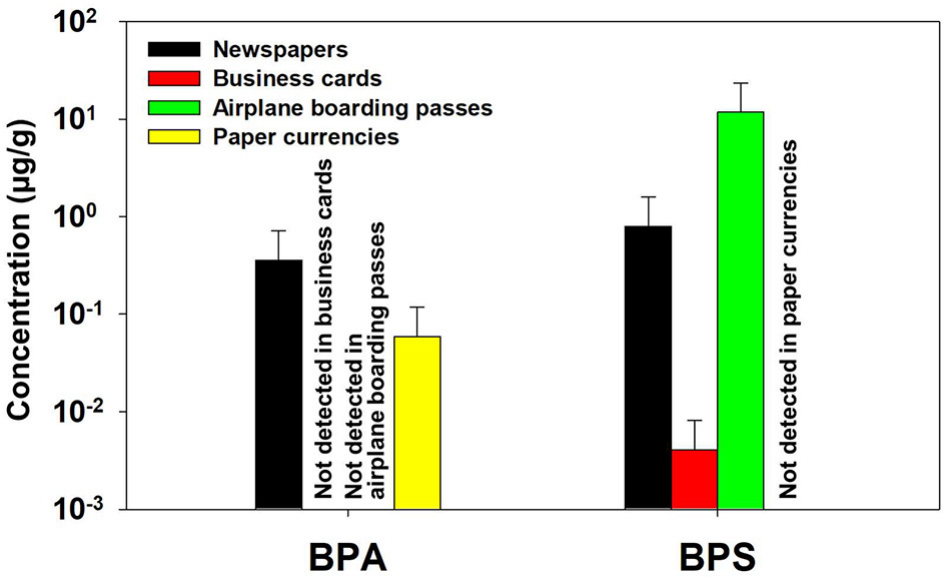
Mean concentrations (μg/g) of BPA and BPS in non-receipt papers.

The concentrations of bisphenol analogs were also measured in other paper products, and the results are shown in Figure 2 and Supplementary Table S6. Among target compounds, only BPA and BPS were detected, while BPF, BPAP, BPP, BPAF, and BPZ were not found in any sample. Overall, ΣBP concentrations in these paper products were 2–4 orders of magnitude lower than those observed in thermal receipts.

BPA and BPS were detected in all newspaper samples, with both compounds present at mean concentrations of 0.36 and 0.80 μg/g for BPA and BPS, respectively. The presence of both BPA and BPS in newspaper samples could be attributable to their high recycling rate among various types of paper-based products. This may be attributable to the high recycling rate of paper products, as previous studies have reported elevated BPA levels in recycled paper, indicating cross-contamination during recycling processes (42–44). Four out of five business card samples contained BPA only, which may reflect the use of recycled paper containing residual BPA and/or the presence of BPA in surface coatings or printing inks used in non-thermal applications (4). No bisphenol analogs were detected in the café coupons, possibly because these products are less likely to be printed on recycled paper or to come into contact with thermal receipts. BPA was detected in 36% of the paper currency samples at very low concentrations (mean 0.06 μg/g), which is likely attributable to secondary contamination through physical contact with thermal receipts during circulation and handling (32, 38). In contrast, airplane boarding passes are printed on thermal paper. In these samples, only BPS was consistently detected (mean: 11.8 μg/g), suggesting that BPS is predominantly used as the color developer in this type of thermal paper.

### 3.4 Estimated dermal exposure dose of bisphenol analogs via handling paper products

Exposure doses to bisphenol analogs through dermal absorption via handling papers are summarized in Table 3. The estimated mean, median, and 95th percentile daily intakes of ΣBP through dermal absorption from handling thermal receipts were 118, 113, and 260 ng/day, respectively, for general populations of Korea. For occupationally exposed groups, the estimated intakes of ΣBP were 4,370, 2,240, and 16,700 ng/day for mean, median, and 95th percentile, respectively, in large retail chains; 11,700, 11,700, and 19,500 ng/day for mean, median, and 95th percentile, respectively, in small local stores; 9,870, 8,700, and 25,100 ng/day for mean, median, and 95th percentile, respectively, in public service facilities. For other paper products, including newspapers, business cards, airplane boarding passes, and paper currencies, the estimated dermal exposure dose was consistently low and nearly identical across exposure groups. In the general population, the mean, median, and 95th percentile intakes of ΣBP were 1.82, 1.72, and 3.20 ng/day, respectively. Similar results were observed in occupational groups, with mean, median, and 95th percentile intakes of 1.85, 1.72, and 3.32 ng/day across large retail chains, small local stores, and public service facilities. These findings indicate that these paper products contributed only marginally to overall bisphenol exposure, with little variation between general and occupational populations. The detailed estimated dermal exposure dose for paper products other than receipts is presented in Supplementary Table S7.

**Table 3:** Estimated dermal exposure dose (ng/day; rounded values) via handling of papers by the general population and occupationally exposed groups and estimated daily intake (ng/kg bw/day).

**Compounds**	**General population exposure**			**Occupational exposure**								
				**Large retail chains**			**Small local stores**			**Public service facilities**		
	**Mean**	**Median**	**95th percentile**	**Mean**	**Median**	**95th percentile**	**Mean**	**Median**	**95th percentile**	**Mean**	**Median**	**95th percentile**
**Thermal receipt papers (ng/day)**												
BPA	118	113	260	4,370	2,240	16,700	11,700	11,700	19,500	9,870	8,700	25,100
BPS	0.12	NC^a^	0.94	12.0	NC	66.1	3.95	NC	7.08	18.5	NC	80.1
ΣBP	119	113	262	4,410	2,240	16,700	11,700	11,700	19,600	9,930	8,700	25,200
**Other paper products (ng/day)**												
BPA	1.73	1.63	3.06	1.76	1.63	3.19	1.76	1.63	3.19	1.76	1.63	3.19
BPS	0.09	0.09	0.14	0.09	0.09	0.14	0.09	0.09	0.14	0.09	0.09	0.14
ΣBP	1.82	1.72	3.20	1.85	1.72	3.32	1.85	1.72	3.32	1.85	1.72	3.32
**Estimated daily intake (ng/kg body weight/day)**												
BPA	1.91	1.82	4.18	69.6	35.7	265	186	186	310	157	139	399
BPS	0.003	0.001	0.02	0.19	0.001	1.06	0.06	0.001	0.12	0.30	0.001	1.28
ΣBP	1.92	1.83	4.22	70.3	35.7	266	186	187	312	158	139	402
Exposure percentage from receipt (%)	98	99	99	>99	>99	>99	>99	>99	>99	>99	>99	>99

^a^NC, not calculated due to concentrations below limit of quantification.

**Figure 3:**
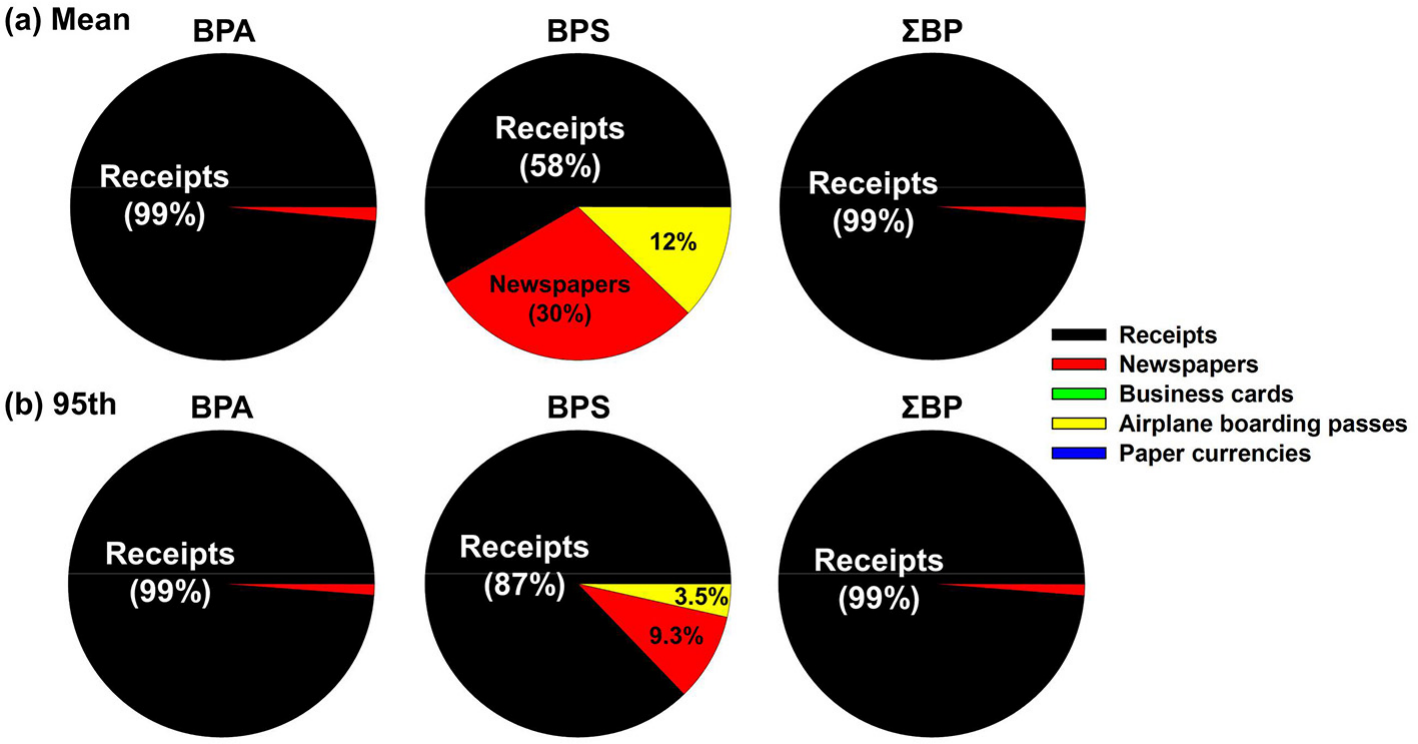
Relative compositional profiles (%) of each type of papers for the estimated daily intake of bisphenol analogs with normal exposure scenario (**a**, mean) and high exposure scenario (**b**, 95th percentile) by general population.

The estimated daily intakes were calculated based on a body weight (bw) of 62.8 kg for adults [aged of 18–74 years, both sexes; (45)] and represent the sum of DEDs from thermal receipts and paper products. The estimated median daily intake of ΣBP was 1.83 ng/kg bw/day for the general population. For occupational groups, the estimated values were 35.7 ng/kg bw/day in large retail chains; 187 ng/kg bw/day in small local stores; and 139 ng/kg bw/day in public service facilities. When compared to other exposure sources for adults in Korea, the estimated intake from handling papers for the general population (1.83 ng/kg bw/day) was higher than that from house dust ingestion [0.81 ng/kg bw/day; (46)], similar to drinking water (3.56 ng/kg bw/day; 2), and approximately 20 times lower than dietary exposure [40.0 ng/kg bw/day; (47)]. In occupational groups, dermal exposure doses associated with handling receipts and other paper products were substantially higher than those from house dust and drinking water, and in some cases comparable to or higher than dietary exposure, suggesting elevated exposure levels for cashiers and similar workers. Lee et al. (1) reported that urinary BPA concentrations in cashiers doubled at post-shift compared to pre-shift levels, directly linking occupational receipt handling to acute increases in internal exposure. Thus, our findings highlight the need for comprehensive assessments of bisphenol exposure and associated adverse health effects among occupationally receipt-handling workers. Figure 3 further illustrates the relative contribution of each paper type to estimated daily intake in the general population. For BPA, thermal receipts overwhelmingly dominated exposure, accounting for nearly all intake (>98% in both mean and 95th percentile scenarios), while other paper products contributed only trace amounts. In contrast, for BPS, although receipts remained the largest contributor, newspapers, and airplane boarding passes also accounted for non-negligible shares under the mean exposure scenario, highlighting the broader distribution of BPS across paper matrices. When combined (ΣBP), receipts still explained over 98% of total exposure, reaffirming their role as the primary source for DEDs of bisphenol analogs through paper handling.

To further contextualize our dermal exposure estimates, they were compared with the EU exposure assessment conducted for the restriction of BPA in thermal paper (48). In that assessment, dermal internal exposure to BPA from thermal paper was estimated using probabilistic and deterministic approaches, yielding a realistic-case estimate of 154 ng/kg bw/day for workers and a selected reasonable worst-case value of approximately 400 ng/kg bw/day, while consumer reasonable worst-case estimates ranged from 31 to 88 ng/kg bw/day. Although the exposure estimates reported by ECHA were generally higher than those obtained in the present study, this difference can be attributed to substantial differences in exposure assumptions and model structure. The ECHA assessment assumed continuous and extensive skin contact with thermal paper and conservative assumptions regarding substance transfer to the skin, whereas the present study estimated dermal exposure based on discrete handling events using measured bisphenol concentrations in paper products. Consequently, occupational exposure estimates in this study (35.7–187 ng/kg bw/day) were lower than or comparable to those reported by ECHA. These findings indicate that differences in dermal exposure estimates across studies are primarily driven by exposure scenario assumptions rather than absorption parameters alone.

The European Food Safety Authority (EFSA) recently reduced the tolerable daily intake (TDI) for BPA to 0.2 ng/kg bw/day (from 4 μg/kg bw/day proposed in 2015), primarily in response to newly evidenced toxicological effects, particularly on immune system (49). Applying this revised benchmark, the DEDs of BPA by paper handling in our study exceeded the TDI of the EFSA for the general population, with occupational groups surpassing it by even larger margins (Table 3). Although this TDI is specific to BPA, accumulating evidence represents that bisphenol analogs target overlapping endocrine endpoints, indicating that reliance on a BPA-only benchmark may underestimate mixture-related risks (19). Therefore, cumulative risk assessment approaches that account for the integrated action of bisphenol analogs acting on common toxicological endpoints are warranted, rather than evaluating BPA alone (34). Furthermore, substitution does not necessarily reduce exposure risks; for instance, higher BPS levels have been observed in children attending “eco-friendly” kindergartens compared with conventional ones (3). These findings suggest that efforts to reduce exposure of bisphenols have achieved only partial success. Nonetheless, short-term dietary interventions have demonstrated notable reductions in urinary BPA levels among both mothers and children (50). In occupational exposure groups, preventive measures, such as wearing gloves or minimizing direct contact with receipts, have also provided measurable benefits in lowering internal exposure (1). Alongside regulatory reforms, public education on practical strategies to limit exposure remains essential for mitigating health risks associated with both BPA and its analogs.

### 3.5 Study limitations and further perspectives

Although the thermal receipt samples analyzed in this study were collected in 2015, corresponding to an early phase of BPA substitution prior to the formal ban in Korea, this timing represents a critical transitional period rather than a methodological limitation. During this period, BPA remained the dominant color developer globally; however, our results reveal that substitution was already occurring in a non-uniform manner across business types. Large retail chains showed earlier and more pronounced adoption of BPA alternatives, whereas small local stores continued to rely heavily on BPA-based receipts, resulting in substantially higher estimated occupational exposures. This business-type-specific heterogeneity has not been sufficiently addressed in previous studies, which have largely focused on national or occupational averages. Although subsequent regulatory actions and market responses have further diversified thermal paper color developers, meaning that absolute exposure levels reported here may not reflect current conditions, the present dataset provides an essential baseline for interpreting post-regulatory trends. In particular, it enables future assessments to evaluate not only overall reductions in bisphenol concentrations but also changes in exposure disparities across retail environments. These findings indicate that market-driven factors such as retailer scale and supply chain characteristics can strongly influence exposure patterns independently of formal regulation, and they highlight the continued relevance of business-type-resolved analyses for understanding chemical substitution dynamics and informing more equitable risk management strategies for bisphenol analogs. Future studies should therefore incorporate more recent samples, expand analytical coverage to emerging developers, and apply longitudinal monitoring aligned with regulatory milestones to assess the effectiveness of BPA restrictions and potential unintended consequences of substitute chemicals.

## 4 Conclusions

This study demonstrated the widespread occurrence of bisphenol analogs in paper products collected in Korea, with thermal receipts identified as the dominant source of dermal exposure. BPA was the predominant compound in most receipts, while BPS represented the main substitute in a subset of products, and other bisphenols were detected only at trace levels consistent with impurities or background contamination. Concentrations of ΣBP in thermal receipts were several orders of magnitude higher than those in other paper products, and compositional analysis revealed distinct usage patterns associated with different color developers. Notably, marked differences in bisphenol profiles and exposure levels were observed across business types, indicating that substitution from BPA to alternative developers occurred earlier and more consistently in large retail chains than in small local stores. Thermal receipts accounted for over 98% of the estimated daily dermal intake of ΣBP, and occupational groups, particularly workers in small local stores, exhibited substantially higher exposure levels than the general population. Estimated BPA intakes via paper handling exceeded the revised EFSA tolerable daily intake, underscoring potential health concerns for receipt-handling workers. Given the shared endocrine-disrupting properties of bisphenol analogs, risk assessments focusing solely on BPA may underestimate cumulative exposure and mixture-related effects. Overall, this study highlights the importance of considering market heterogeneity and business-type-specific practices when evaluating human exposure to bisphenols from thermal paper products.

## Data Availability

The original contributions presented in the study are included in the article/Supplementary material, further inquiries can be directed to the corresponding author.
