# Supplementary material for: Occurrence and human exposure assessment of bisphenol analogues in various paper products from Korea
Source: Front Public Health. 2026 Feb 4;14:1748104. doi: 10.3389/fpubh.2026.1748104 (PMC12913405; doi:10.3389/fpubh.2026.1748104)
Supplement: Supplementary file 1 [file Data_Sheet_1.docx]

**[Supplementary material]**

**Occurrence and human exposure assessment of bisphenol analogues in various paper products from Korea**

Mangong Shin^1^, Jae-Eun Lim^1^, Sori Mok^1^, Chunyang Liao^2^, Hyo-Bang Moon^*,1^

^1^ Department of Marine Science and Convergence Technology, College of Engineering Sciences, Hanyang University, Ansan 15588, Republic of Korea

^2^ State Key Laboratory of Environmental Chemistry and Ecotoxicology, Research Center for Eco‑Environmental Sciences, Chinese Academy of Sciences, Beijing 100085, China

Figures: 6

Tables: 7

Table S1. Target bisphenol analogues in this study.

| **Chemical structure** | **Chemical name** | **Abbreviation** | **CAS** | **Chemical formula** | **MW** |
| --- | --- | --- | --- | --- | --- |
| 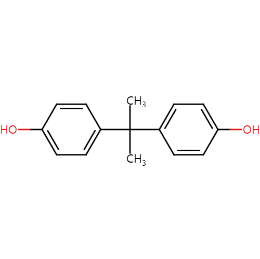 | 2,2-Bis(4-hydroxyphenyl)propane | BPA | 80-05-7 | C_15_H_16_O_2_ | 228.29 |
| 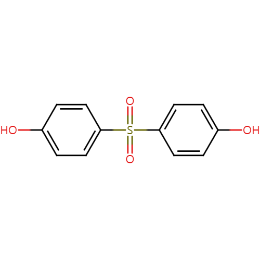 | Bis(4-hydroxyphenyl)sulfone | BPS | 80-09-1 | C_12_H_10_O_4_S | 250.27 |
| 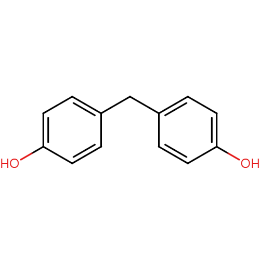 | Bis(4-hydroxyphenyl)methane | BPF | 620-92-8 | C_13_H_12_O_2_ | 200.24 |
| 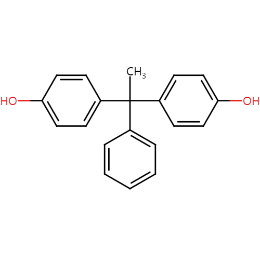 | 1,1-Bis(4-hydroxyphenyl)-1-phenylethane | BPAP | 1571-75-1 | C_20_H_12_O_2_ | 290.36 |
| 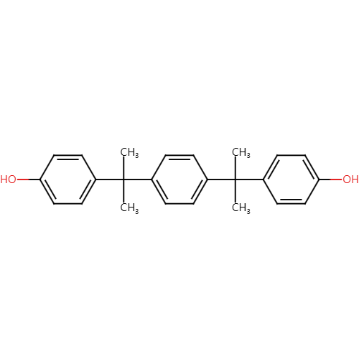 | 4-[2-[4-[2-(4-hydroxyphenyl)propan-2-yl]phenyl]propan-2-yl]phenol | BPP | 2167-51-3 | C_24_H_26_O_2_ | 346.46 |
| 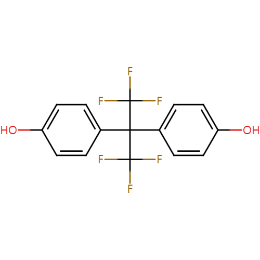 | 4,4′-(1,1,1,3,3,3-Hexafluoropropane-2,2-diyl)diphenol | BPAF | 1478-61-1 | C_15_H_10_F_6_O_2_ | 336.23 |
| 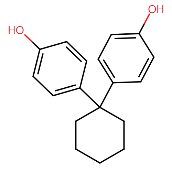 | 1,1-Bis(4-hydroxyphenyl)cyclohexane | BPZ | 843-55-0 | C_18_H_20_O_2_ | 268.35 |

Table S2. Selected multiple reaction monitoring (MRM) transitions and optimized potentials of the target compounds.

| **Compound** | **Precursor**  **(m/z)** | **Product**  **(m/z)** | **DP^a^ (volt)** | **EP^b^ (volt)** | **CE^c^ (volt)** | **CXP^d^ (volt)** |
| --- | --- | --- | --- | --- | --- | --- |
| BPA | 227 | 211 | -75 | -10 | -26 | -9 |
| BPS | 249 | 108 | -90 | -10 | -34 | -3 |
| BPF | 199 | 77 | -85 | -10 | -32 | -7 |
| BPAP | 289 | 273 | -120 | -10 | -32 | -9 |
| BPP | 345 | 330 | -120 | -10 | -40 | -13 |
| BPAF | 335 | 265 | -90 | -10 | -32 | -9 |
| BPZ | 267 | 173 | -65 | -10 | -38 | -11 |
| ^13^C_12_-BPA | 239 | 223 | -85 | -10 | -26 | -7 |
| ^13^C_12_-BPS | 261 | 114 | -95 | -10 | -38 | -7 |

^a^ DP: declustering potential.

^b^ EP: entrance potential.

^c^ CE: collision energy.

^d^ CXP: collision cell exit potential.

Table S3. Summary of matrix effects, regression coefficients (R^2^), and limit of quantification (LOQ) for the seven target bisphenol analogues.

| **Compound** | **Matrix effect (%)**  **10 ng/mL**  **(mean ± SD^a^)** | **Matrix effect (%)**  **100 ng/mL**  **(mean ± SD)** | **R^2^** | **LOQ (ng/g)** |
| --- | --- | --- | --- | --- |
| BPA | 103 ± 1.8 | 112 ± 2.2 | 0.9991 | 5.0 |
| BPS | 105 ± 0.8 | 94 ± 0.5 | 0.9986 | 0.5 |
| BPF | 105 ± 1.0 | 107 ± 13 | 0.9986 | 25 |
| BPAP | 98 ± 1.5 | 106 ± 10 | 0.9978 | 0.5 |
| BPP | 99 ± 1.1 | 113 ± 7.7 | 0.9986 | 2.5 |
| BPAF | 106 ± 0.6 | 67 ± 11 | 0.9987 | 2.5 |
| BPZ | 94 ± 1.0 | 106 ± 4.7 | 0.9987 | 2.5 |

^a^ SD: standard deviation.

Table S4. Exposure factors used for dermal exposure dose calculation.

| **Parameter** | **Value(s)** | **Unit** | **Applied for** | **Reference** |
| --- | --- | --- | --- | --- |
| *k* | 21522.4 | ng/s |  | Biedermann et al., 2010 |
| HF^c^ | 2 (GP^a^),  150 (Occ^b^) | times/day | thermal receipts | Liao and Kannan, 2011b |
|  | 2 (GP),  20 (Occ) | times/day | paper currencies | Liao and Kannan, 2011a |
|  | 5 | times/day | business cards, airplane boarding passes, café coupons | Liao and Kannan, 2011b |
|  | 15 | times/day | newspapers | Fan et al., 2015 |
| HT^d^ | 5 | s | all papers except newspaper | Liao and Kannan, 2011b |
|  | 60 | s | newspapers | Fan et al., 2015 |
| AF^e^ | 25 | % | BPA and other bisphenol analogues | Raele et al., 2021 |
|  | 0.4 | % | BPS |  |

^a^ GP: general population.

^b^ Occ: occupationally exposed individuals.

^c^ HF: handling frequency.

^d^ HT: handling time.

^e^ AF: dermal absorption fraction.


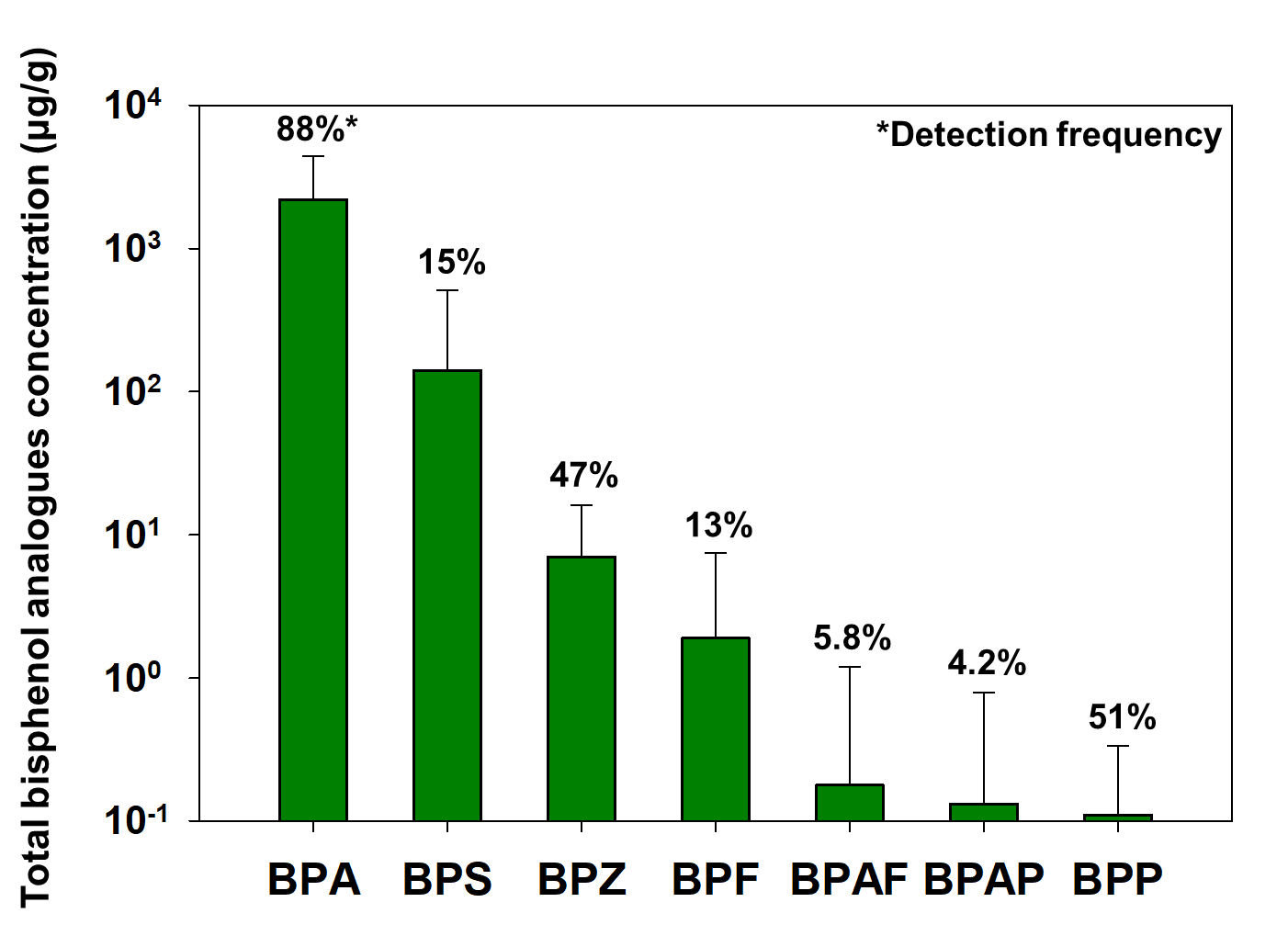


Figure S1. Mean concentrations and detection frequencies of seven bisphenol analogues in thermal receipt papers.


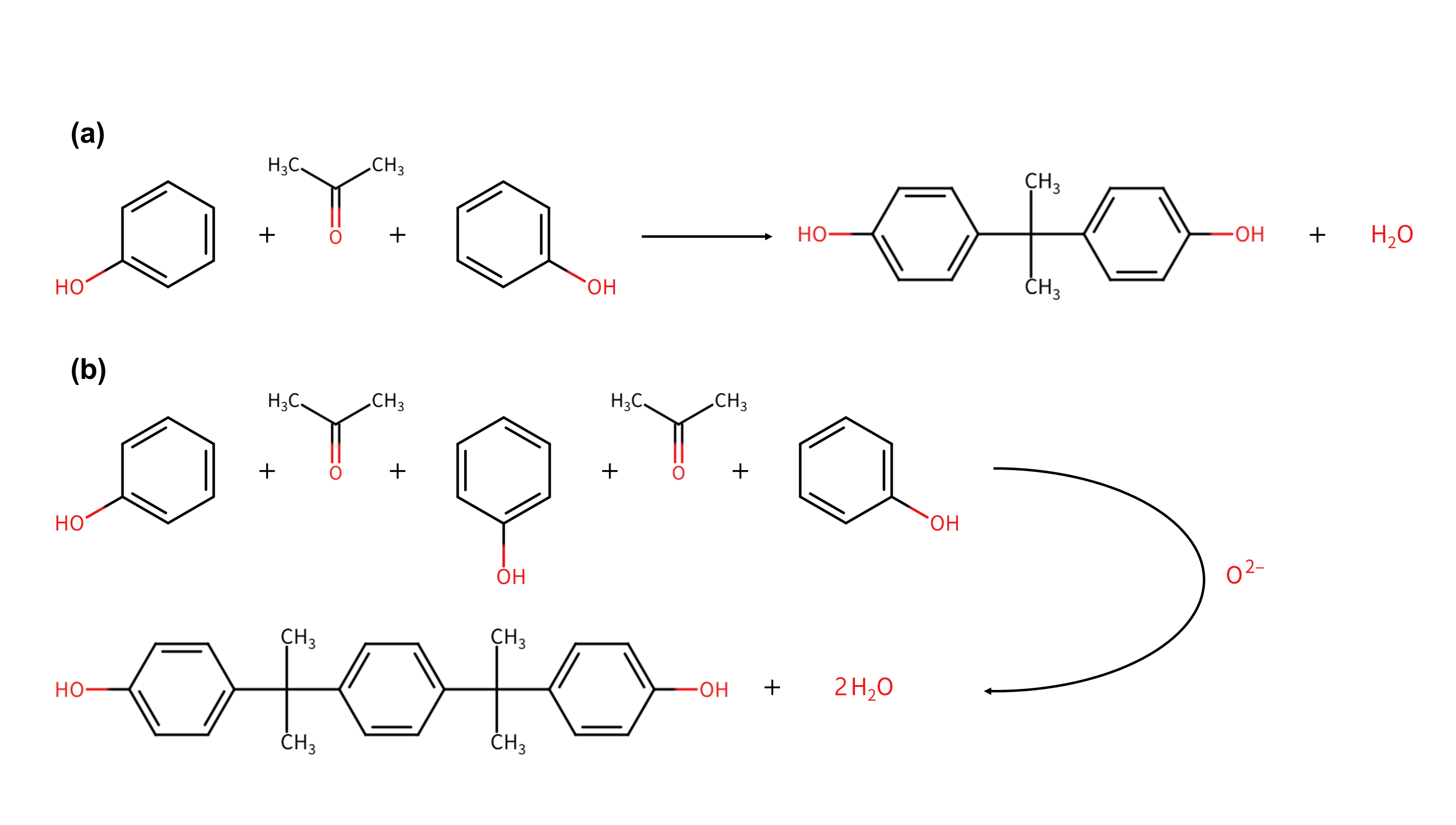


Figure S2. Proposed reaction pathway for BPA synthesis and potential formation of BPP as a byproduct: (a) synthesis of BPA from phenol and acetone; (b) unintended formation of BPP as an impurity during BPA production.


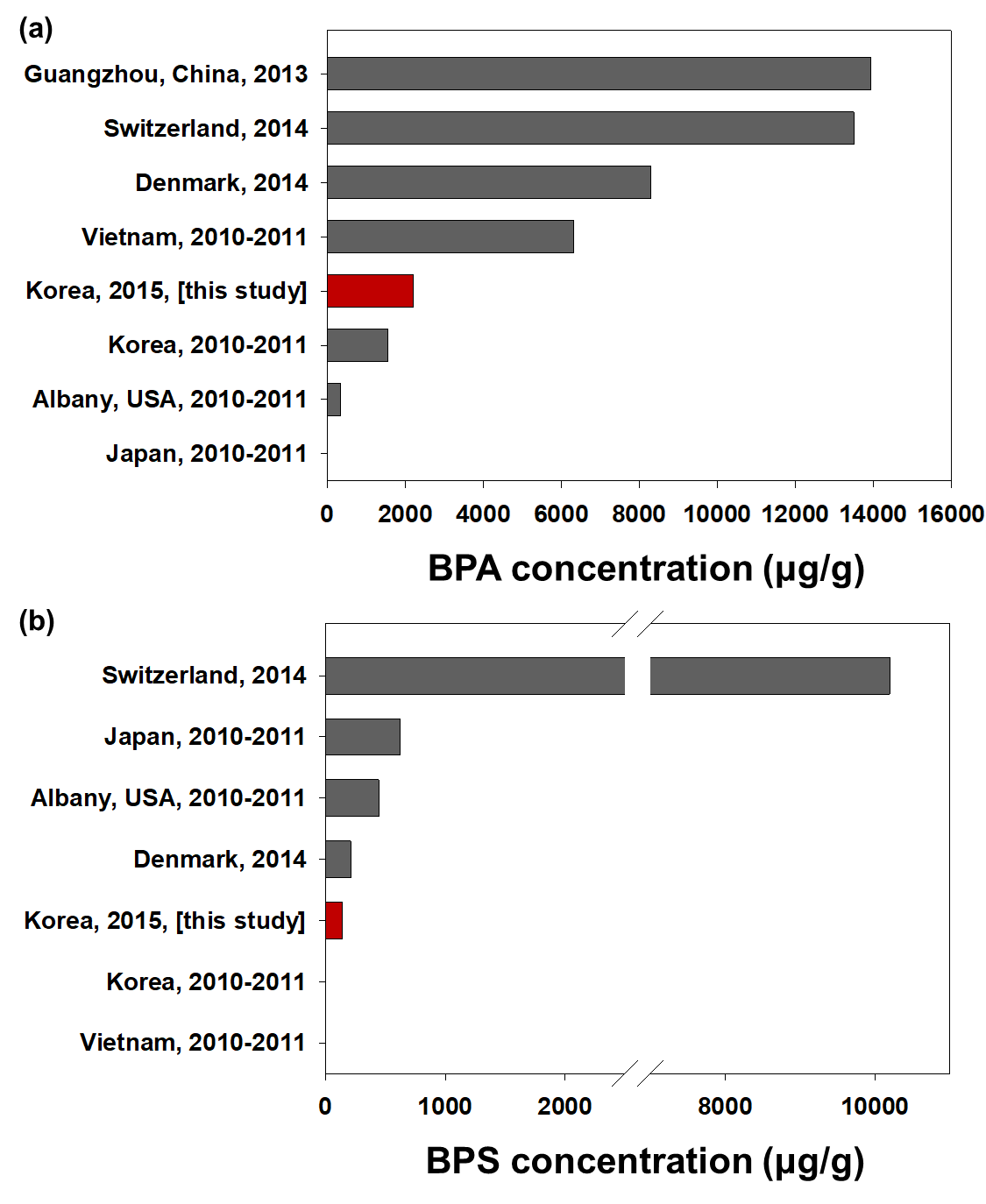


Figure S3. Comparison of mean concentrations of (a) BPA and (b) BPS in thermal receipt papers between this study and previous studies.


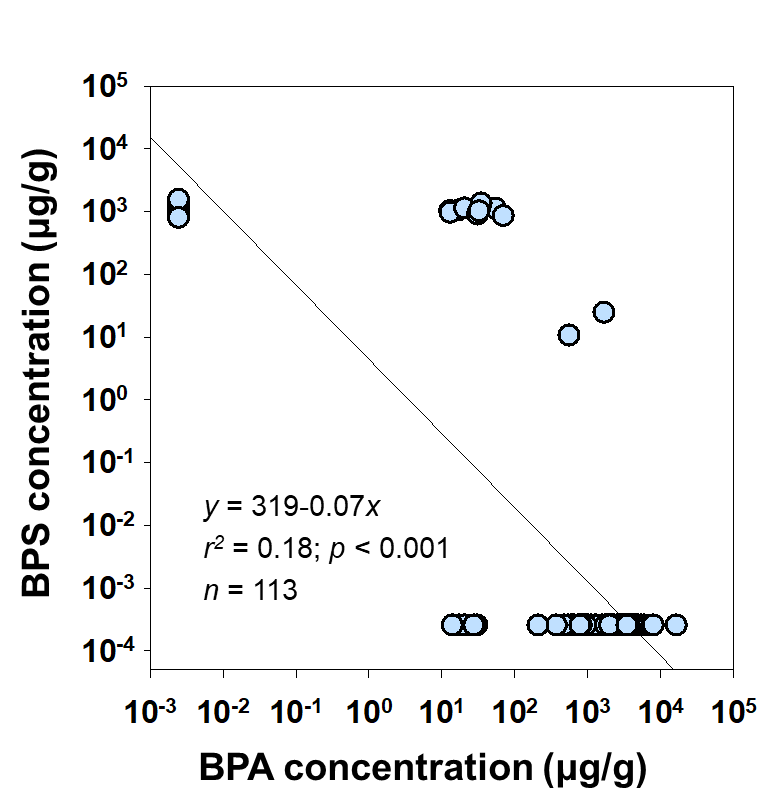


Figure S4. Scatter plot showing the relationship between BPA and BPS concentrations in thermal receipt papers (*n* = 113).

Table S5. Occurrence and concentrations of bisphenol analogues (μg/g) in thermal receipt by cluster.

|  | BPA | BPS | BPF | BPAP | BPP | BPAF | BPZ | ΣBP |
| --- | --- | --- | --- | --- | --- | --- | --- | --- |
|  | **Cluster 1 (*n* = 93)** | | | | | | | |
| DF^a^ (%) | 100 | 2.2 | 12 | 2.2 | 52 | 7.5 | 46 | 100 |
| Mean | 2830 | 0.37 | 1.90 | 0.08 | 0.11 | 0.23 | 7.07 | 2840 |
| Median | 2780 | <LOQ^b^ | <LOQ | <LOQ | 0.02 | <LOQ | <LOQ | 2790 |
| Max | 16800 | 24.1 | 32.2 | 4.85 | 1.53 | 9.71 | 64.0 | 16900 |
| SD^c^ | 2110 | 2.71 | 5.73 | 0.59 | 0.24 | 1.14 | 9.58 | 2120 |
|  | **Cluster 2 (*n* = 16)** | | | | | | | |
| DF (%) | 56 | 100 | 13 | 19 | 56 | 0 | 50 | 100 |
| Mean | 18.2 | 1060 | 1.53 | 0.50 | 0.11 | -^d^ | 7.00 | 1080 |
| Median | 13.2 | 1030 | <LOQ | <LOQ | 0.08 | - | 6.06 | 1050 |
| Max | 71.0 | 1520 | 14.1 | 3.32 | 0.63 | - | 16.7 | 1520 |
| SD | 22.0 | 197 | 4.24 | 1.09 | 0.17 | - | 7.30 | 194 |
|  | **Cluster 3 (*n* = 5)** | | | | | | | |
| DF (%) | 80 | 0 | 20 | 0 | 60 | 0 | 100 | 100 |
| Mean | 19.3 | - | 2.01 | - | 0.15 | - | 14.5 | 36.2 |
| Median | 20.9 | - | <LOQ | - | 0.11 | - | 12.7 | 43.6 |
| Max | 30.9 | - | 10.0 | - | 0.45 | - | 18.6 | 49.2 |
| SD | 12.1 | - | 4.49 | - | 0.19 | - | 3.62 | 15.9 |
|  | **Outlier (*n* = 1)** | | | | | | | |
| Concentration | - | - | 16.9 | - | 0.28 | - | - | 17.2 |

^a^ DF: detection frequency; ^b^ LOQ: limit of quantification; ^c^ SD: standard deviation; ^d^ -: not reported for compounds with no detection.


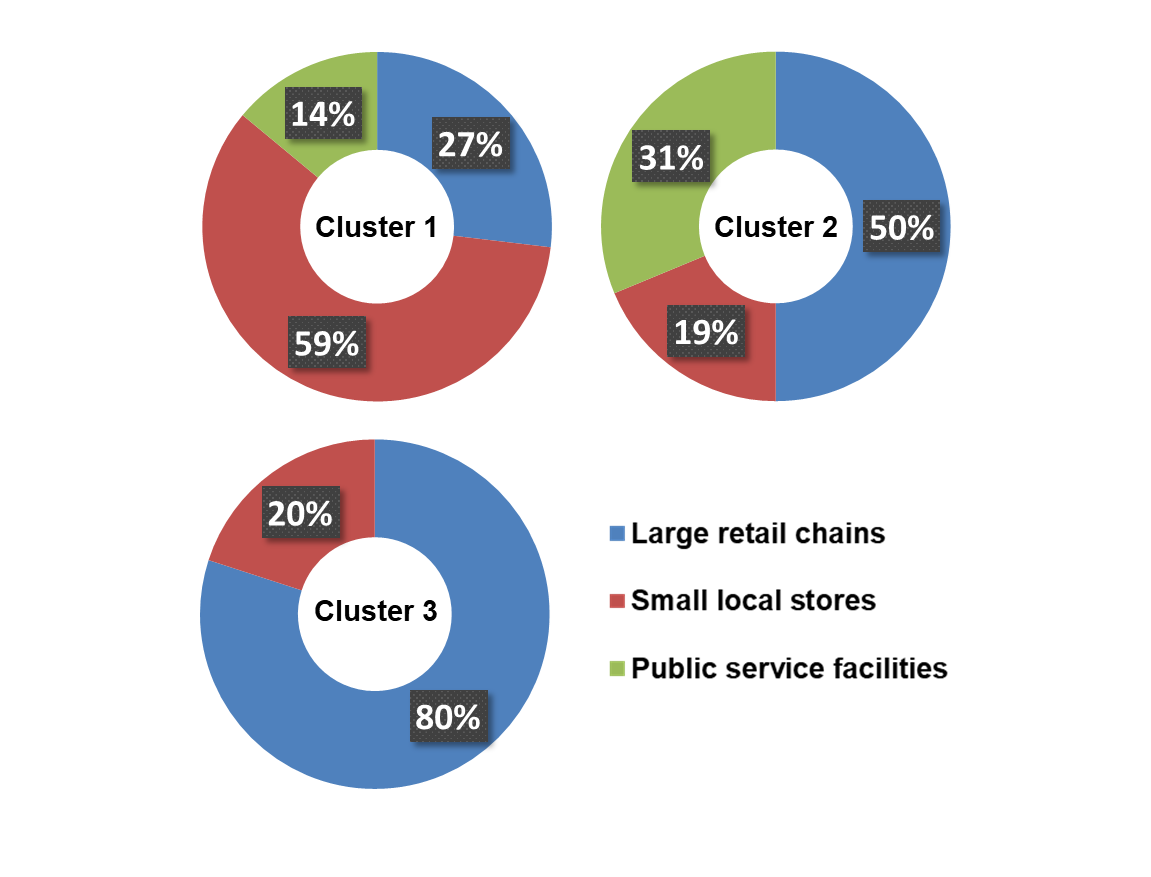


Figure S5. Cluster composition of thermal receipt samples by market type.


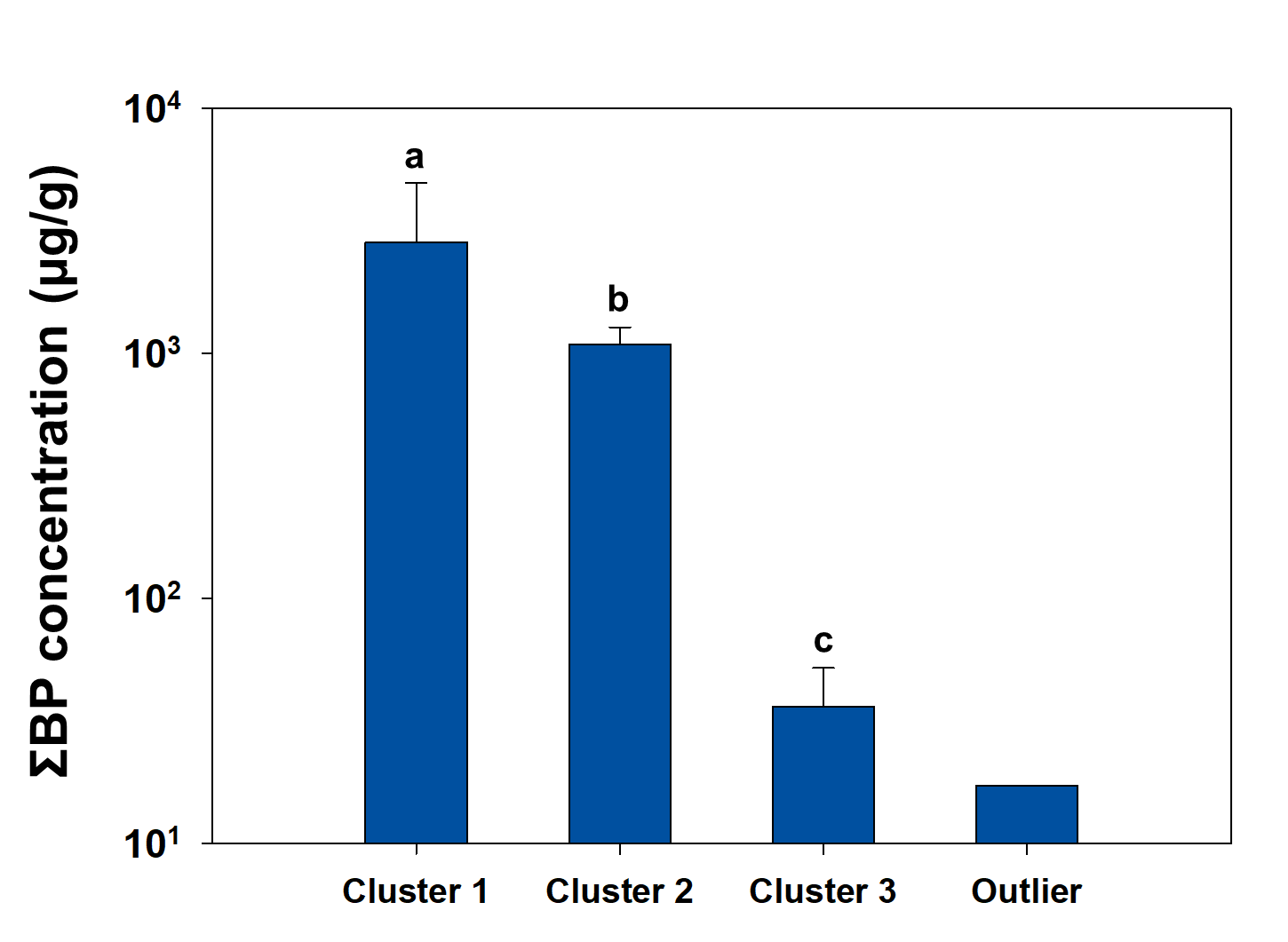


Figure S6. Total concentrations of bisphenol analogues (μg/g) in thermal receipt samples by n-MDS-derived cluster. Bars not sharing the same letter (a–c) are significantly different (*p* < 0.05). The outlier refers to a single receipt sample that was not assigned to clusters 1–3.

Table S6. Occurrence and concentrations of bisphenol analogues (μg/g) in other paper products.

|  | BPA | BPS | ΣBP | |
| --- | --- | --- | --- | --- |
| **Newspapers (*n* = 5)** | | | |  |
| DF^a^ (%) | 100 | 100 | 100 | |
| Mean | 0.36 | 0.80 | 1.15 | |
| Median | 0.34 | 0.66 | 1.03 | |
| Max | 0.65 | 1.39 | 2.03 | |
| SD^b^ | 0.25 | 0.39 | 0.52 | |
| **Business cards (*n* = 5)** | | | |  |
| DF (%) | 0 | 80 | 80 | |
| Mean | -^c^ | 0.004 | 0.004 | |
| Median | - | 0.001 | 0.001 | |
| Max | - | 0.01 | 0.01 | |
| SD | - | 0.006 | 0.006 | |
| **Airplane boarding passes (*n* = 6)** | | | |  |
| DF (%) | 0 | 100 | 100 | |
| Mean | - | 11.8 | 11.8 | |
| Median | - | 16.5 | 16.5 | |
| Max | - | 17.6 | 17.6 | |
| SD | - | 8.06 | 8.06 | |
| **Paper currencies (*n* = 5)** | | | |  |
| DF (%) | 36 | 0 | 36 | |
| Mean | 0.06 | - | 0.06 | |
| Median | <LOQ^d^ | - | <LOQ | |
| Max | 0.36 | - | 0.36 | |
| SD | 0.11 | - | 0.11 | |

^a^ DF: detection frequency; ^b^ SD: standard deviation; ^c^ -: not reported for compounds with no detection; ^d^ LOQ: limit of quantification.

Table S7. Estimated dermal exposure doses (ng/day; rounded values) of bisphenol analogues from paper products other than receipts.

|  | BPA | BPS | ΣBP | |
| --- | --- | --- | --- | --- |
| **Newspapers (*n* = 5)** | | | |  |
| Mean | 1.73 | 0.06 | 1.79 | |
| 95th percentile | 3.05 | 0.10 | 3.15 | |
| **Business cards (*n* = 5)** | | | |  |
| Mean | NC^a^ | 8.74x10^-6^ | 8.74x10^-6^ | |
| 95th percentile | NC | 2.63x10^-5^ | 2.63x10^-5^ | |
| **Airplane boarding passes (*n* = 6)** | | | |  |
| Mean | NC | 0.03 | 0.03 | |
| 95th percentile | NC | 0.04 | 0.04 | |
| **Paper currencies (*n* = 5)** | | | |  |
| Mean | 0.003 | NC | 0.003 | |
| 95th percentile | 0.01 | NC | 0.01 | |

^a^ NC: not calculated due to concentrations below limit of quantification.

**References**

Biedermann, S., Tschudin, P., Grob, K., 2010. Transfer of bisphenol A from thermal printer paper to the skin. Anal. Bioanal. Chem. 398, 571−576.

Fan, R., Zeng, B., Liu, X., Chen, C., Zhuang, Q., Wnag, Y., Hu, M., Lv, Y., Li, J., Zhou, Y., Lin, Z.Y.W., 2015. Levels of bisphenol-A in different paper products in Guangzhou, China, and assessment of human exposure via dermal contact. Evnviron. Sci. Process. Impacts 17, 667−673.

Liao, C., Kannan, K., 2011a. High levels of bisphenol A in paper currencies from several countries, and implications for dermal exposure. Environ. Sci. Technol. 45, 6761−6768.

Liao, C., Kannan, K., 2011b. Widespread occurrence of bisphenol A in paper and paper products: implications for human exposure. Environ. Sci. Technol. 45, 9372−9379.

Reale, E., Vernez, D., Hopf, N.B., 2021. Skin absorption of bisphenol A and its alternatives in thermal paper. Ann. Work Expo. Health. 65, 206–218.
